# Equipment-free, unsupervised high intensity interval training elicits significant improvements in the physiological resilience of older adults

**DOI:** 10.1186/s12877-022-03208-y

**Published:** 2022-06-28

**Authors:** Tanvir S Sian, Thomas B Inns, Amanda Gates, Brett Doleman, Joseph J Bass, Philip J Atherton, Jonathan N Lund, Bethan E Phillips

**Affiliations:** 1 MRC-Versus Arthritis Centre for Musculoskeletal Ageing Research and NIHR Nottingham Biomedical Research Centre, School of Medicine, University of Nottingham, Derby, UK; 2 Department of Surgery and Anaesthesia, Royal Derby Hospital, University Hospitals of Derby and Burton, Derby, UK

**Keywords:** Exercise, Supervision, Cardiorespiratory fitness, HIIT, Cardiometabolic

## Abstract

**Background:**

Reduced cardiorespiratory fitness (CRF) is an independent risk factor for dependency, cognitive impairment and premature mortality. High-intensity interval training (HIIT) is a proven time-efficient stimulus for improving both CRF and other facets of cardiometabolic health also known to decline with advancing age. However, the efficacy of equipment-free, unsupervised HIIT to improve the physiological resilience of older adults is not known.

**Methods:**

Thirty independent, community-dwelling older adults (71(SD: 5) years) were randomised to 4 weeks (12 sessions) equipment-free, supervised (in the laboratory (L-HIIT)) or unsupervised (at home (H-HIIT)) HIIT, or a no-intervention control (CON). HIIT involved 5, 1-minute intervals of a bodyweight exercise each interspersed with 90-seconds recovery. CRF, exercise tolerance, blood pressure (BP), body composition, muscle architecture, circulating lipids and glucose tolerance were assessed at baseline and after the intervention period.

**Results:**

When compared to the control group, both HIIT protocols improved the primary outcome of CRF ((via anaerobic threshold) mean difference, L-HIIT: +2.27, H-HIIT: +2.29, both *p* < 0.01) in addition to exercise tolerance, systolic BP, total cholesterol, non-HDL cholesterol and *m. vastus lateralis* pennation angle, to the same extent. There was no improvement in these parameters in CON. There was no change in diastolic BP, glucose tolerance, whole-body composition or HDL cholesterol in any of the groups.

**Conclusions:**

This is the first study to show that short-term, time-efficient, equipment-free, HIIT is able to elicit improvements in the CRF of older adults irrespective of supervision status. Unsupervised HIIT may offer a novel approach to improve the physiological resilience of older adults, combating age-associated physiological decline, the rise of inactivity and the additional challenges currently posed by the COVID-19 pandemic.

**Trial registration:**

This study was registered at clinicaltrials.gov and coded: NCT03473990.

**Supplementary Information:**

The online version contains supplementary material available at 10.1186/s12877-022-03208-y.

## Background

Cardiovascular disease is the most common cause of death worldwide, accounting for nearly one third of deaths each year [1]. Physical inactivity and advancing age are each associated with an increased risk of cardiovascular disease [2], with just a quarter of older adults in the UK meeting the *minimum* recommended activity levels needed to maintain health [3]. Considering the ongoing COVID-19 pandemic, physical activity levels amongst older adults are reported to have reduced further due to national lockdowns and shielding behaviours [4], lessening their physiological resilience to health-related challenges.

Cardiorespiratory fitness (CRF) is not only the “gold-standard” measure of physical fitness [5], but is also a recognised marker of cardiovascular health, and prognostic marker of both cardiovascular disease [6] and premature mortality [7, 8]. Known to decline with advancing age [9], reductions in CRF have been suggested to be due to declines in mitochondrial quality and reductions in maximal ATP production and aerobic respiration rate [10, 11]. Exemplifying the impact of physical activity on CRF, sedentary adults with lower CRF have a mortality risk that is 4.5 times higher than those that exercise regularly [12], with reduced CRF a stronger risk factor for all-cause mortality than hypertension, smoking and diabetes [13].

Beyond CRF, another important risk factor for premature mortality in older adults is sarcopenia; the age-associated loss of muscle mass and function [14, 15]. Exacerbated by physical inactivity [15], sarcopenia is a precursor to both falls and frailty syndromes, and is directly associated with lower CRF [16]. Another age-associated change in body composition which is also associated with physical inactivity is excess accumulation of body fat. Associated with cardiovascular disease and metabolic dysfunction (i.e., insulin resistance and dyslipidaemia), obesity in older age not only impacts morbidity and mortality, but also impacts quality of life and independence maintenance [17].

Exercise is a proven strategy to improve CRF, muscle mass and metabolic status in older adults [12, 18, 19], with aerobic [20] and resistance exercise training [21] each eliciting distinct favourable adaptations. However, high-intensity interval training (HIIT) appears to confer improvements commonly associated with both aerobic and resistance exercise training [22], whilst also addressing a commonly cited barrier to exercise uptake and adherence, “lack of time” [23]. Beyond “time”, older adults also frequently cite their age and “poor health” as barriers to exercise [24], despite a significant body of literature evidencing the benefits of all types of exercise training (aerobic: [25, 26], resistance: [21, 27], HIIT: [28, 29]) for even the oldest old [30]. Further, the *Generation 100* randomised control trial has demonstrated that older adults can perform both aerobic exercise and HIIT without strict supervision [31, 32], suggesting that home-based, time-efficient HIIT without the need for specialist equipment may prove a promising strategy to improve the physiological resilience of older adults, especially at a time when access and desire to attend specialist exercise facilities (i.e., gyms and community classes) may be at an all-time low.

We have previously demonstrated that 4-weeks equipment-free, unsupervised HIIT can elicit comparable improvements in the CRF of middle-aged adults when compared to supervised, cycle ergometer-based HIIT [33], and that supervision status does not impact the efficacy of HIIT for improving CRF in young adults [34]. However, to date, no study has investigated the impact of short-term, equipment-free, unsupervised HIIT for improving multiple parameters of physiological resilience in older adults.

## Methods

### Subject characteristics

Thirty independent community-dwelling older (71(SD = 5) years) adults (14 male) who were disability free and able to perform activities of daily living without support or assistive devices, and who were not engaged in a formal exercise regime (defined as 2 or more sessions per week) were recruited to this study. Further exclusion criteria were taken from the American Thoracic Society/American College of Chest Physicians, and the Preoperative Exercise Testing and Training Society [35, 36] safety guidelines for exercise testing, which include, but are not limited to: numerous cardiac abnormalities, hemodynamic compromise, untreated resting hypertension, lower limb thrombus, pulmonary oedema, embolus or significant hypertension and orthopaedic or cognitive impairment leading to inability to perform the exercise or cooperate, respectively. After health screening and provision of informed consent, participants were randomised to each intervention group using random permuted blocks of: (i) laboratory (supervised) HIIT (L-HIIT), (ii) home (unsupervised) HIIT (H-HIIT) and (iii) a no-intervention control (CON). Ethical approval for the study was obtained from the University of Nottingham Faculty of Medicine and Health Sciences Research Ethics Committee (C16122016). The study was registered at clinicaltrials.gov (NCT03473990) and all aspects of the study were performed in accordance with the Declaration of Helsinki.

### Assessment sessions

Assessment sessions were conducted 72 h before and 72 h after a 4-week intervention period. Participants were asked to fast from 2200 h the preceding night and to attend the research unit at 0900 h. Measures of height and weight were taken and resting BP was measured in accordance with established guidelines [37]. Before a two-hour oral glucose tolerance test (OGTT) was conducted [38], body composition was determined by dual-energy x-ray absorptiometry (DXA; Lunar Prodigy 2, GE Medical Systems, Buckinghamshire, UK). Baseline blood samples from the OGTT were subject to clinical chemistry analysis to determine cholesterol profiles. Plasma insulin concentrations from all OGTT samples were measured using an ultrasensitive enzyme linked immunosorbent assay (ELISA; Mercodia AB, Uppsala, Sweden), with blood glucose measured on a near-patient glucose analyser (YSI Life Sciences, Ohio, USA). During the OGTT, muscle architecture (fibre pennation angle (PA), muscle thickness (MT) and fascicle length (FL)) was assessed using B-mode ultrasound (MyLab™50; Esaote, Genoa, Italy) as previously described [39, 40].

After provision of a standardised meal and rest period, a cardiopulmonary exercise testing (CPET) was performed in accordance with the aforementioned guidelines [35, 36] on a cycle ergometer (Lode Corival, Lode BV, Groningen, Netherlands). Breath-by-breath data was collected using an inline gas analysis system (ZAN 680, nSpire Health, Colorado, USA). Each test began with a 3-minute period of observed rest on the cycle ergometer followed by a 2-minute warm-up of unloaded cycling. Participants were encouraged to exercise to volitional exhaustion, with termination criteria as per safety guidelines [35, 36]. VO_2_ peak was defined as the highest VO_2_ attained during the test with anaerobic threshold (AT) defined using a combination of the modified V-slope and ventilatory equivalents methods by two blinded experienced assessors [41].

### Interventions

**Fig. 1:**
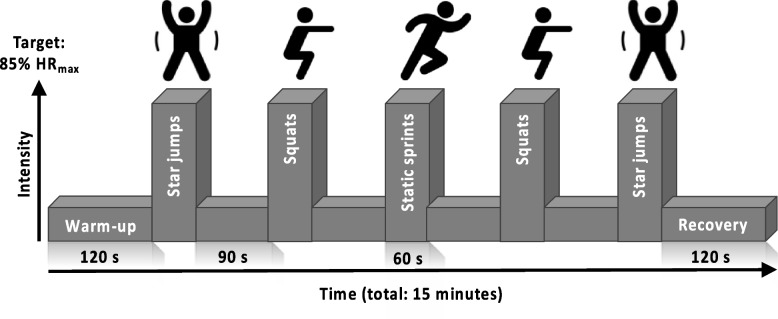
Schematic representation of the high intensity interval training (HIIT) protocol perform with and without supervision. Abbreviations: HR_max_, age-predicted maximum heart rate (220-age); s, seconds

The HIIT protocol used was based on previous work demonstrating the efficacy of this temporal protocol to improve CRF when performed on a cycle ergometer [33, 42] and with bodyweight-based exercises [33, 34]. All sessions began with a warm-up period of two minutes walking on-the-spot before five, one-minute high-intensity efforts were performed, each interspersed by 90 s of active recovery. Following the final effort, a 2-minute recovery period concluded each session. Exercises were performed in a pyramid formation, designed to provide motivation to maintain intensity during fatigue (i.e., in efforts 4 and 5) (Fig. 1).

All participants assigned to a HIIT group attended the research unit for a HIIT introductory session before starting training. This session ensured that participants were comfortable with the temporal profile of the sessions, were coached for proper form during each exercise, and showed H-HIIT participants how to record engagement with each session. The aim for each effort during every session was to achieve a heart rate > 85% age-predicted maximum [43].

All participants were asked to maintain their habitual level of physical activity and dietary intake for the duration of the study. Participants in both HIIT groups completed 3 sessions each week for 4 weeks. L-HIIT participants completed this under one-to-one supervision at the research unit, with verbal encouragement throughout each session. H-HIIT participants completed their sessions at home with no supervision or feedback. Participants in the H-HIIT group were given a printed resource outlining the HIIT protocol and a fingertip pulse oximeter (iMDK, MediSave, UK) to assess HR at the end of each effort. Training adherence (number of sessions) and compliance (% of sessions where the target heart rate threshold was achieved) for the H-HIIT group was determined via instructed self-report using a training diary and the fingertip pulse oximeter, with attendance and HR recorded by the research team for the L-HIIT group.

### Statistical analysis

Based on CRF data from a previous study [33], an *a priori* power calculation using the R pwr package, based on an improvement in CRF from 15.28 to 18.23ml/kg/min, provides an effect size of 1.12, which for n = 10 per group provides a power of greater than 80%. Descriptive statistics are reported as number (%) or mean (SD). Change between groups was determined using ANCOVA with baseline values as the continuous covariates [44]. Within group analysis for pre to post intervention change was performed using repeated measures t-tests. Effect estimates are presented as mean differences (MD) with 95% confidence intervals (CI). Due to lack of normality, equality of variance and heteroscedasticity for pennation angle, these data were log transformed. Analyses were conducted using Stata Version 16 (StataCorp LLC, TX, USA). The level of significance was set at *p* < 0.05.

## Results

### Participant characteristics

Thirty independent community-dwelling older adults were recruited to this study. Groups were matched for age, BMI, CRF, BP and cholesterol at baseline (Table 1).

**Table 1:** Participant baseline characteristics

	L-HIIT (*n* = 10)	H-HIIT (*n* = 10)	CON (*n* = 10)
**Age (years)**	70 (5)	71 (4)	71 (7)
**Male / Female**	5/5	3/7	6/4
**BMI**	26 (3)	25 (3)	26 (1)
**AT**	13.40 (4)	14.90 (6)	14.27 (3)
**VO**_**2**_**peak**	26.37 (8)	25.47 (8)	28.05 (4)
**SBP**	127 (14)	126 (10)	128 (11)
**DBP**	73 (7)	68 (12)	77 (7)
**Total Cholesterol**	5.1 (1.5)	5.7 (0.9)	5.1 (0.7)
**Co-morbidities**			
− BPH	1	-	-
− HTN	2	2	2
− Asthma	-	1	1
− Depression	-	1	-
− OA	-	1	3
− HCL	-	1	1
− Hypothyroidism	-	-	1
**Medications**			
− Alpha-blocker	1	-	-
− ACE-inhibitor	2	1	1
− Statins	-	1	1
− Ca + channel blocker	-	1	1
− SSRI	-	1	-
− Thyroxine	-	-	1
− Beta-2 adrenergic receptor agonist	-	1	1

Data depicts mean (SD) or for co-morbidities and medication class. Abbreviations: L-HIIT, laboratory (supervised) high intensity interval training; H-HIIT, home-based (unsupervised) HIIT; CON, no-intervention control group; BMI, body mass index (kg/m^2^); AT, anaerobic threshold (ml/kg/min); VO_2_ peak, peak oxygen uptake (ml/kg/min); SBP, systolic blood pressure (mmHg); DBP, diastolic BP; BPH, benign prostatic hyperplasia; HTN, hypertension; HCL, hypercholesterolaemia; OA, osteoarthritis; ACE, angiotensin converting enzyme; Ca+, calcium; SSRI, serotonin re-uptake inhibitor. There were no significant differences between groups in any numerical parameter

### Study adherence

After randomisation, no participants were lost to follow-up. There were no adverse safety events reported in any group. Two participants (1 H-HIIT, 1 CON) did not have a post intervention DXA scan. Training adherence and compliance was 100% for both HIIT groups.

### Cardiorespiratory fitness and exercise tolerance

**Fig. 2:**
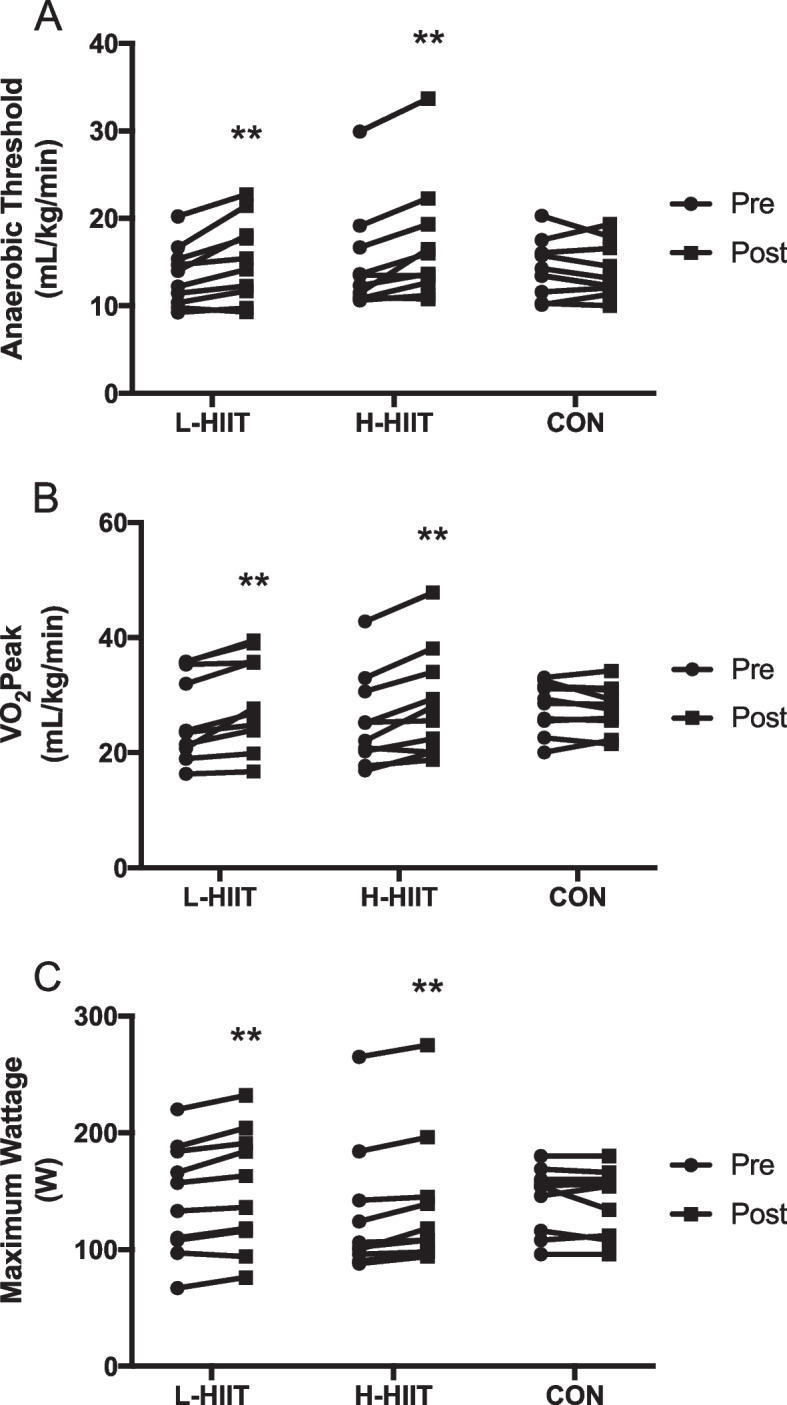
Anaerobic threshold (**A**), VO_2_peak (**B**) and maximum wattage (**C**) in older individuals before and after laboratory (L-HIIT; *n* = 10) or home-based (H-HIIT; *n* = 10) high intensity interval training (HIIT) or a no intervention control (CON; *n* = 10) period. **= *p* < 0.01 versus pre-intervention

There was a significant increase in AT in L-HIIT (MD + 2.27 (0.57 to 3.98) ml/kg/min; *p* = 0.007) and H-HIIT (MD + 2.29 (0.59 to 4) ml/kg/min; *p* = 0.006) when compared to CON, with no difference in change between the HIIT groups (MD -0.02; 95% CI -1.74 to 1.7; *p* = 1.0) (Fig. 2 A, Supplementary Information Table S1). Similarly, there was a significant increase in VO_2_peak in L-HIIT (MD + 3.05 (0.81 to 5.29) ml/kg/min; *p* = 0.005) and H-HIIT (MD + 3.45 (1.19 to 5.7) ml/kg/min; *p* = 0.002) when compared to CON, with no difference in change between the HIIT groups (MD -0.4 (-2.63 to 1.84) ml/kg/min; *p* = 0.96) (Fig. 2B, Table S1).

Exercise tolerance, determined as maximum wattage (Wmax), increased in both L-HIIT (MD + 10.64 (2.9 to 18.38) W; *p* = 0.005) and H-HIIT (MD + 10.57 (2.98 to 18.16) W; *p* = 0.005) when compared to CON, with no difference in change between the HIIT groups (MD + 0.07 (-7.68 to 7.82) W; *p* = 1) (Fig. 2 C, Table S1).

### Cardiometabolic status

**Fig. 3:**
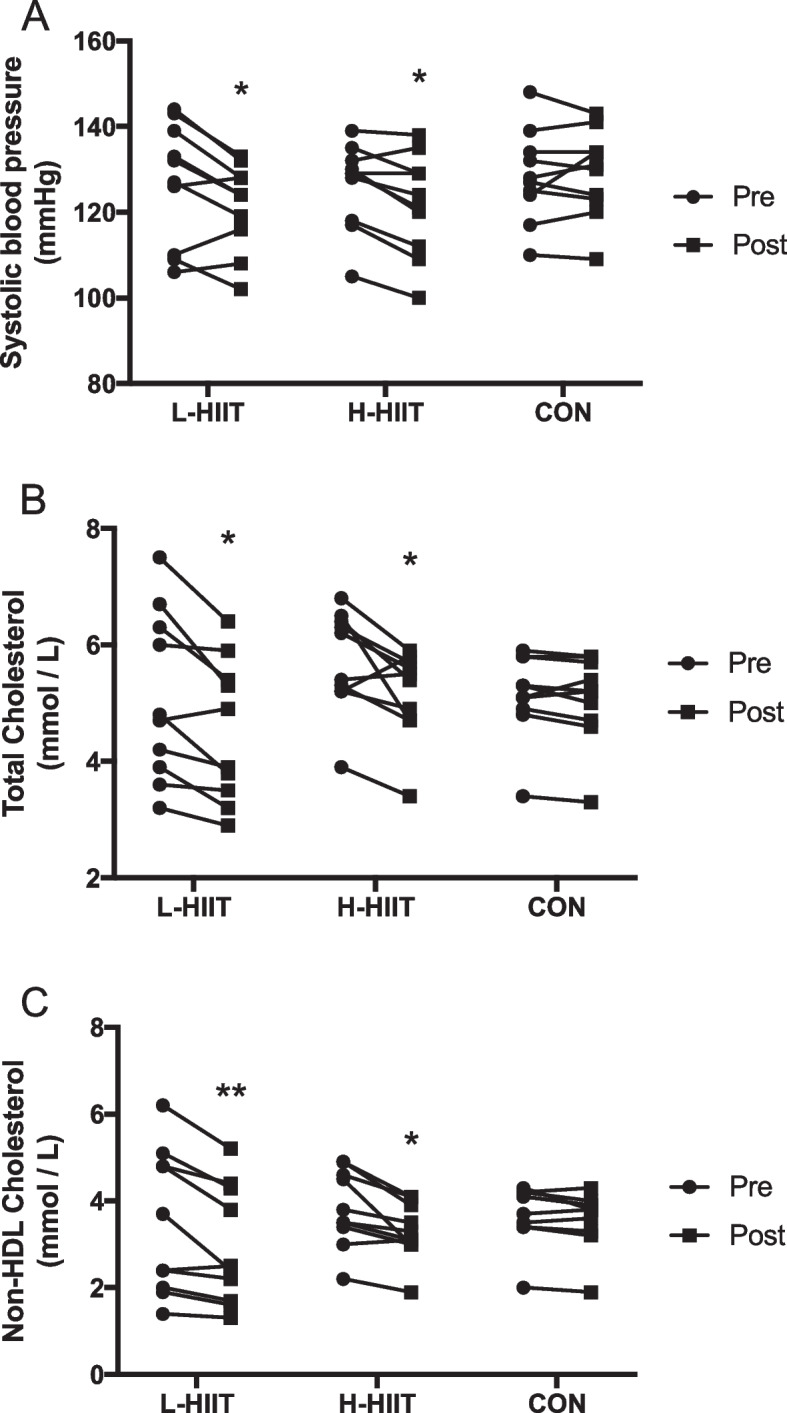
Systolic blood pressure (**A**), total cholesterol (**B**) and non-HDL cholesterol (**C**) in older individuals before and after laboratory (L; n = 10) or home-based (H; n = 10) high intensity interval training (HIIT) or a no intervention control (CON; *n* = 10) period. *=*p* < 0.05, **= *p* < 0.01 versus pre-intervention

There was a significant reduction in resting systolic BP in L-HIIT (MD -6 (-12 to -1) mmHg; *p* = 0.02) and H-HIIT (MD -6 (-11 to 0) mmHg; *p* = 0.05) when compared to CON, with no difference in change between the HIIT groups (MD -1 (-6 to 5) mmHg; *p* = 0.97) (Fig. 3 A, Table S1). There were no significant changes in resting diastolic BP in any group (Table S1).

There was a significant reduction in both total (L-HIIT (MD -0.51 (-1.01 to -0.01) mmol/L; *p* = 0.04) and H-HIIT (MD -0.46 (-0.87 to -0.05) mmol/L; *p* = 0.05)) (Fig. 3B) and non-HDL (L-HIIT (MD -0.46 (-0.81 to -0.11) mmol/L; *p* = 0.008) and H-HIIT (MD -0.38 (-0.73 to -0.03) mmol/L; *p* = 0.03) (Fig. 3 C) cholesterol in L-HIIT and H-HIIT when compared to CON, with no difference in change between the HIIT groups (Table S1). HDL cholesterol did not change in any group (Table S1). Neither glucose nor insulin area-under-the-curve (AUC) during the OGTT, or the homeostatic model assessment of insulin resistance (HOMA-IR) changed in any group (Table S1).

### Body composition and muscle architecture

**Fig. 4:**
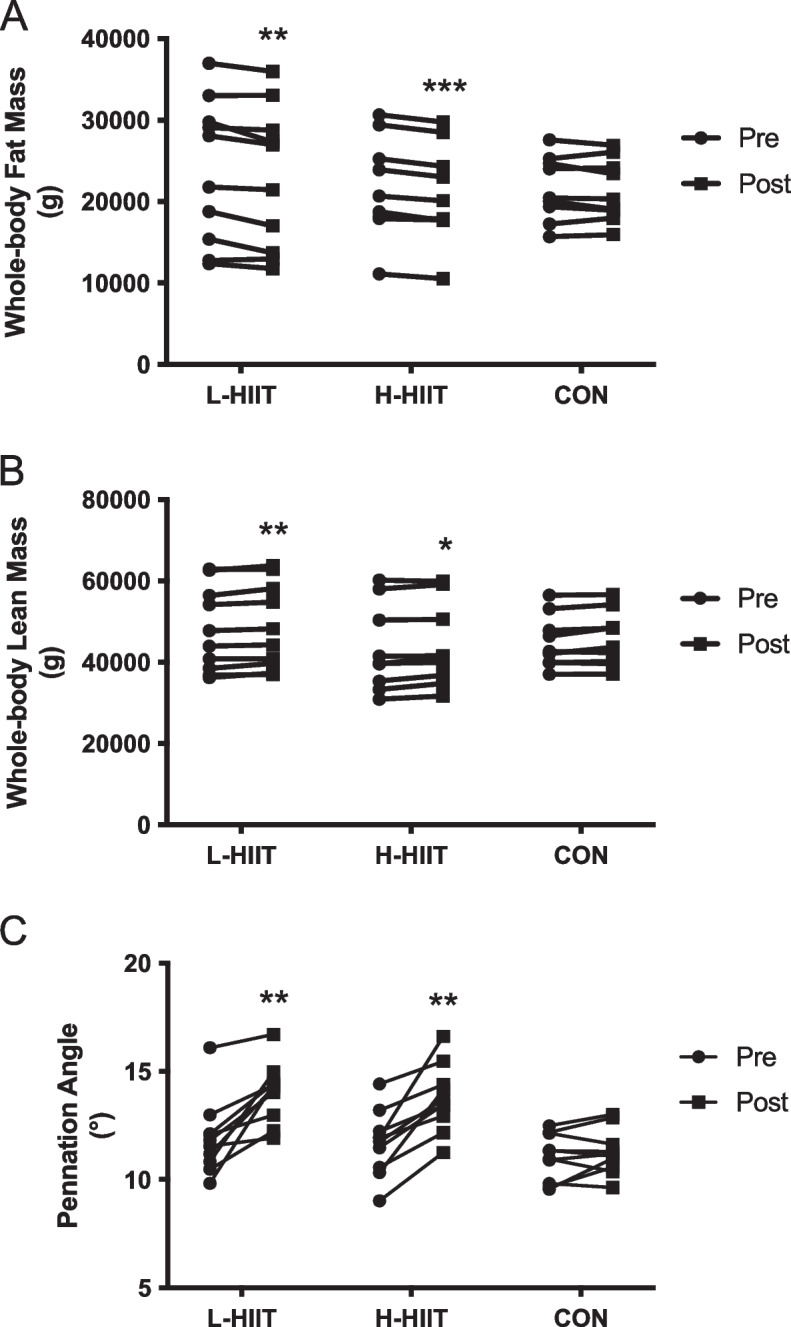
Whole-body fat (**A**) and lean (**B**) mass and *m. vastus lateralis* pennation angle (**C**) in older individuals before and after laboratory (L; *n* = 10) or home-based (H; *n* = 10) high intensity interval training (HIIT) or a no intervention control (CON; n = 10) period. **= *p* < 0.01, ***=*p* < 0.01 versus pre-intervention

There was a significant decrease in total fat mass in L-HIIT and H-HIIT (Fig. 4 A, Table S1), although only the reduction in L-HIIT was different to CON (Table S1). Percentage body fat decreased in both L-HIIT and H-HIIT, but these changes were not significantly different to CON (Table S1). Similarly, whole-body lean mass increased in both L-HIIT and H-HIIT, but these changes were not significantly different to CON (Fig. 4B, Table S1).

There was a significant increase in *m. vastus lateralis* PA in L-HIIT (MD + 1.95 (0.72 to 3.17) º; *p* = 0.001) and H-HIIT (MD + 1.8 (0.59 to 3.01) º; *p* = 0.002) when compared to CON, with no difference in change between the HIIT groups (MD + 0.14 (-1.05 to 1.34) º; p = 0.99) (Fig. 4 C, Table S1). Neither *m. vastus lateralis* MT nor FL changed in any group (Table S1).

## Discussion

Whilst the positive effects of HIIT on CRF in older adults are well-established [20, 42, 45], to our knowledge, this is the first study to show that just four-weeks, time-efficient, equipment-free HIIT is able to elicit improvements in the CRF, body composition and cardiovascular health of older adults. Further, these benefits are also achievable via unsupervised, home-based exercise. The rapidity of these improvements demonstrate the potential for this exercise regime to be used in time-constrained clinical settings commonly associated with advancing age (i.e., prior to cancer surgery [42, 45]), as well as to improve the general health and physiological resilience of an ageing population [4].

In addition to being able to elicit improvements in a number of physiological parameters key to optimal health across the life-course, the HIIT regime employed in this study also addresses the issue of “lack of time”, a commonly cited barrier to exercise for older adults [46, 47]. The improvements reported herein were achieved with a total time commitment of < 50 min per week; one third of the current guidelines for moderate continuous exercise [48]. That the benefits achieved were largely indistinguishable between the two HIIT groups, contests the need for supervision to optimise the benefits of exercise [49], a notion supported by previous work reporting on middle-aged adults [50]. Although there appears to be no data reporting the impact of HIIT supervision in older adults, in young [34] and middle-aged [33] adults, and in adults with type 1 diabetes [51] and heightened cardiovascular disease risk [52], supervision was not shown to enhance adaptation. It should however be noted that in the latter two studies telehealth monitoring and feedback was employed, compared to the self-report of adherence and absence of feedback for the home-based group in this study. Conversely, improvements in functional balance were reported as superior with supervision during strength and coordination training [53, 54], suggesting that the influence of supervision is likely impacted by the type of exercise training and/or the assessed outcomes.

Another key benefit to the HIIT regime employed in this study is that no facilities or equipment are required, removing two further barriers (access to facilities and reticence to exercise in public [23, 55, 56]) to exercise for older adults. In addition, our HIIT protocol only included three exercises, addressing concerns about complexity [49], each of which are easily adaptable for those with, for example, joint restrictions which commonly occur in older age [57]. It is our belief, that in a pragmatic roll-out of equipment-free, unsupervised HIIT to improve the health of older adults, any exercise which can be performed safely to raise heart to > 85% of age-predicted maximum could be substituted into the temporal regime reported herein.

That we observed significant improvements in AT, VO_2_peak and exercise tolerance adds to the growing body of literature reporting that HIIT is safe and effective for improving CRF in older adults [42, 58–60]. In addition, HIIT is able to elicit these gains in a shorter time-frame than that commonly reported for aerobic exercise training [61]. For example, the improvements we report herein, using an equipment free protocol (irrespective of supervision) are not dissimilar to those previously seen in older adults with HIIT of the same temporal profile but on a specialised cycle ergometer; findings which have been replicated in comorbid octogenarians [30]. Not only are improvements in CRF associated with reduced cardiovascular disease risk and all-cause mortality in older adults [7], but the magnitude of improvement observed in this study is above the reported minimum clinically important difference in AT (1.5 ml/kg/min) to reduce post-operative risk [62]. This finding exemplifies the broad potential of this equipment-free HIIT regime to improve the physiological resilience of older adults in different situations.

Aligned to the reduction in CVD risk associated with improved CRF [7], reductions in both SBP and cholesterol (total and non-HDL) are also associated with an reduced risk of all-cause mortality in older adults [63]. Our finding of reduced resting SBP, irrespective of supervision, is in agreement with previous HIIT studies of older adults, albeit with most of these based having a longer training period [18], greater time-commitment [64] and supervision [45]. In addition, the magnitude of our reductions in resting SBP are superior to those reported with 3-months, time-intense aerobic exercise training in older adults [65], and similar to that elicited by 6-weeks HIIT on a cycle ergometer [20]. Our reduction in cholesterol aligns with previous suggestions that higher-intensity exercise is required to reduce non-HDL cholesterol effectively [66], further supporting the notion that HIIT may be an alternative option to traditional exercise training modalities to reduce cardiovascular disease risk in older adults.

To our surprise, despite a wealth of evidence suggesting that insulin sensitivity can be improved in older adults via HIIT [58, 67, 68], and that HIIT can combat the severity of type 2 diabetes [69, 70], we were not able to show improvement in any marker of insulin sensitivity. This may be due to a longer time period of training being required and/or due to improvements in insulin sensitivity being more pronounced in subjects with existing insulin resistance [71].

Importantly, given the largely inevitable and progressive development of sarcopenia and the relationship between low muscle mass and all-cause mortality, falls, frailty [72] and dependency [73] in older adults, the HIIT regime employed in this study was able to elicit favourable changes in body composition. These changes include increased whole-body lean mass, increased muscle pennation angle, reduced fat mass and reduced body mass index. Muscle pennation angle has been shown to be associated with sarcopenia progression [74] and is an accepted marker of force and contractile capabilities [75] which corresponds to both muscle cross-sectional area and muscle strength [76]. Increased fat mass and obesity are both related to the development of cardiometabolic disorders [77], and as such improvements in these parameters further exemplifies the potential of this intervention for maintaining whole-body health across the life course. It must however be acknowledged that for the majority of these parameters the changes seen in the HIIT groups were not statistically different to that observed in the control group- perhaps owing to a lack of statistical power to determine between group differences. Furthermore, the magnitude of reduction in fat mass seen in this study is not consistent with numerous studies (including those in older adults) [61, 67], where an average 6% reduction was seen with ten weeks of HIIT [78]. That our individuals were only slightly overweight on average, and/or that our HIIT regime was considerably shorter may explain this discrepancy, whilst also highlighting the potential for this regime to have even more impact in older individuals with less favourable body composition profiles [67].

As with all studies, we recognise some limitations to this work. Whilst participants were asked to maintain their current level of physical activity and habitual dietary intake for the duration of the study, this was not measured, and as such alterations in these aspects may have influenced outcomes of this study. In addition, our participants were older community-dwelling volunteers with only well-controlled comorbidities. The ability of multimorbid, frail, dependent and/or very elderly individuals to undertake unsupervised equipment-free HIIT has been poorly studied to date and given the ageing population should be considered in future work. Finally, while our home-based intervention was delivered without supervision or engagement, participants did attend for an in-person instruction session (and so that study-related resources could be provided). As such, future studies considering the ‘roll-out’ of home-based HIIT may wish to explore the impact of remote delivery of the instruction session to facilitate a wholly home-based intervention.

### Conclusions

In conclusion, four-weeks, time-efficient, equipment-free HIIT is able to elicit improvements in the CRF, body composition and cardiometabolic health of older adults irrespective of supervision. This application of HIIT at home may provide a novel approach to increase the physiological resilience of older adults by combating the rise of inactivity and associated poor health in a growing section of society; both of which are magnified by current challenges associated with the COVID-19 pandemic.

## Supplementary Information

**Additional file 1: Supplementary Table 1 (S1).** Assessment parameters before (pre) and after (post) a 4-week period of laboratory (supervised) high-intensity interval training (L-HIIT), home (unsupervised) HIIT (H-HIIT) or a no intervention control (CON) period.

## Data Availability

The datasets from this study are available from the corresponding author on reasonable request.

## Abbreviations

CRF: cardiorespiratory fitness
HIIT: high-intensity interval training
L-HIIT: Lab HIIT, H-HIIT: Home HIIT
CON: control (group)
BMI: body mass index
AT: anaerobic threshold
VO2peak: peak volume of oxygen
SBP: systolic blood pressure
OGTT: oral glucose tolerance test
DXA: dual-energy x-ray absorptiometry
ELISA: enzyme linked immunosorbent assay
PA: pennation angle
MT: muscle thickness
FL: fascicle length
CPET: cardiopulmonary exercise test
HRmax: maximum heart rate
MD: mean difference
CI: confidence interval
Wmax: maximum wattage
BP: bloos pressure
HDL: high-density lipoprotein
AUC: area under-the-curve
HOMA-IR: homeostatic model assessment of insulin resistance
