# Correction: Equipment-free, unsupervised high intensity interval training elicits significant improvements in the physiological resilience of older adults

**DOI:** 10.1186/s12877-022-03488-4

**Published:** 2022-12-01

**Authors:** Tanvir S. Sian, Thomas B. Inns, Amanda Gates, Brett Doleman, Joseph J. Bass, Philip J. Atherton, Jonathan N. Lund, Bethan E. Phillips

**Affiliations:** 1 MRC-Versus Arthritis Centre for Musculoskeletal Ageing Research and NIHR Nottingham Biomedical Research Centre, School of Medicine, University of Nottingham, Derby, UK; 2 Department of Surgery and Anaesthesia, Royal Derby Hospital, University Hospitals of Derby and Burton, Derby, UK

**Correction: BMC Geriatrics 22, 529 (2022)**

**https://doi.org/10.1186/s12877-022-03208-y**

After publication of this article [1], the authors reported that the wrong Supplementary file was originally published with this article; it has now been replaced with the correct file.

The original article [1] has been corrected.

## Supplementary Information

**Additional file 1: Supplementary Table 1 (S1):** Assessment parameters before (pre) and after (post) a 4-week period of laboratory (supervised) high-intensity interval training (L-HIIT), home (unsupervised) HIIT (H-HIIT) or a no intervention control (CON) period.
