# Supplementary material for: Equipment-free, unsupervised high intensity interval training elicits significant improvements in the physiological resilience of older adults
Source: BMC Geriatr. 2022 Jun 28;22:529. doi: 10.1186/s12877-022-03208-y (PMC9238013; doi:10.1186/s12877-022-03208-y)
Supplement: Supplementary file 1 — Additional file 1: Supplementary Table 1 (S1). Assessment parameters before (pre) and after (post) a 4-week period of laboratory (supervised) high-intensity interval training (L-HIIT), home (unsupervised) HIIT (H-HIIT) or a no intervention control (CON) period. [file 12877_2022_3208_MOESM1_ESM.docx]

## SUPPLEMENTARY INFORMATION

**Supplementary Table 1 (S1):** Assessment parameters before (pre) and after (post) a 4-week period of laboratory (supervised) high-intensity interval training (L-HIIT), home (unsupervised) HIIT (H-HIIT) or a no intervention control (CON) period.

|  | **L-HIIT** | | **H-HIIT** | | | **CON** | |
| --- | --- | --- | --- | --- | --- | --- | --- |
|  | **Pre** | **Post** | **Pre** | **Post** | **Pre** | | **Post** |
| **Cardiorespiratory fitness and exercise tolerance** | | | | | | | |
| AT (ml/kg/min) | 13.40 (4) | 15.24 (5)*^ | 14.90 (6) | 16.94 (7)*^ | 14.27 (3) | | 13.94 (3) |
| VO_2_ (ml/kg/min) | 26.37 (8) | 28.98 (8)*^ | 25.47 (8) | 28.42 (9)*^ | 28.05 (4) | | 27.7 (4) |
| W_max_ (W) | 143 (48) | 151.4 (51)*^ | 129.7 (56) | 137.9 (57)*^ | 144 (28) | | 142.1 (28) |
| **Cardiometabolic status** | | | | | | | |
| SBP (mmHg) | 127 (14) | 121 (10)*^ | 126 (10) | 122 (12)*^ | 128 (11) | | 129 (10) |
| DBP (mmHg) | 73 (7) | 69 (7) | 68 (12) | 64 (9) | 77 (7) | | 73 (12) |
| Total Cholesterol | 5.1 (1.5) | 4.5 (1.2)*^ | 5.7 (0.9) | 5.2 (0.8)*^ | 5.1 (0.7) | | 5.1 (0.8) |
| Non-HDL | 3.5 (1.7) | 2.9 (1.4)*^ | 3.8 (0.9) | 3.3 (0.7)*^ | 3.7 (0.7) | | 3.6 (0.7) |
| HDL | 1.6 (0.5) | 1.6 (0.4) | 1.9 (0.4) | 1.9 (0.4) | 1.5 (0.2) | | 1.5 (0.2) |
| Glucose AUC | 894.84 (103) | 895.03 (125) | 920.69 (129) | 915.01 (118) | 956.18 (234) | | 950.12 (213) |
| Insulin AUC | 5284.8 (4176) | 5431.8 (4644) | 6645.5 (2374) | 8409.6 (5410) | 5411.1 (2263) | | 6576.7 (3341) |
| HOMA-IR | 0.98 (0.5) | 0.97 (0.4) | 2.2 (1.9) | 1.3 (1.0) | 1.7 (1.5) | | 2.5 (2.4) |
| **Body composition & muscle architecture** | | | | | | | |
| BMI (kg/m^2^) | 25.95 (3) | 25.77 (3)* | 24.54 (3) | 24.37 (3)* | 25.57 (1) | | 25.48 (1) |
| Lean Mass (g) | 47353.9 (9652) | 48036.0 (9972)* | 38664.1 (6407) | 39496.6 (5972)* | 45615.4 (7163) | | 46070.0 (7268) |
| Fat Mass (g) | 23808.0 (8774) | 22905.5 (8751)* | 21762.0 (6182) | 21064.8 (6015)* | 21596.7 (3981) | | 21421.6 (3845) |
| % Fat | 32.85 (10) | 31.82 (10)* | 33.63 (8) | 32.54 (8)* | 32.53 (6) | | 31.98 (7) |
| PA (degrees) | 11.90 (2) | 14.03 (1)*^ | 11.69 (2) | 13.76 (2)*^ | 11.04 (1) | | 11.26 (1) |
| MT (mm) | 1.94 (0.4) | 2 (0.3) | 1.85 (0.5) | 1.84 (0.3) | 1.89 (0.3) | | 1.96 (0.3) |
| FL (mm) | 7.21 (2) | 7.40 (2) | 6.66 (2) | 6.89 (2) | 6.97 (2) | | 6.90 (2) |

Abbreviations: AT, anaerobic threshold; SBP, systolic blood pressure; DBP, diastolic blood pressure; BMI, body mass index; HDL, high-density lipoprotein; PA, (muscle) pennation angle; MT, muscle thickness; FL, (muscle) fascicle length); AUC, area under the curve (presented as mmol/120 minutes for glucose and uU/ml/120 minutes for insulin); HOMA-IR, homeostatic model assessment of insulin resistance. Data are presented as mean (SD). Analysis via repeated measures t-tests for within group changes, and ANCOVA with baseline values as the continuous covariates for differences in between group changes. *=p<0.05 versus pre-intervention in the same group; ^=p<0.05 versus change in the CON group.
